# iTEARS: a novel platform for using tears as potential disease biomarkers

**DOI:** 10.1097/JS9.0000000000000087

**Published:** 2023-01-18

**Authors:** Abdul Moiz Khan, Syeda Shahnoor

**Affiliations:** a Department of Internal Medicine, Ayub Medical College, Abbottabad; b Department of Internal Medicine, Dow University of Health Sciences, Karachi, Pakistan

*Dear Editor,*

Exosomes are double-membrane lipid bilayer nanoparticles secreted into extracellular spaces by various cells of the body^1^. They can be found in several body fluids such as serum, breast milk, saliva, cerebrospinal fluid, semen, and urine in both physiological and pathological conditions^2^. They have been indicated to have diagnostic applications in terms of both their morphology and miRNA expression. Exosome extraction and assessment are made efficient by the identification of various transport proteins, CD9, CD81, and heat shock protein, all of which are an integral part of exosome composition^3^. Around 4400 distinct proteins have been identified in exosomes using mass spectrophotometry^4^. Much research is being conducted, which focuses on the employment of these vesicles as innovative biomarkers for diagnosis, prognosis, and management of cancer and other diseases^2^.

In comparison with other biofluids, tears, because of their less protein and debris content as compared to blood and its noninvasive collection, are the most appropriate source for exosome isolation. Their easy accessibility for biomarker analysis using the Schirmer test strip greatly boasts its importance^5^. To gain access to the gateway of a multitude of diseases in a drop of tear, successful approaches have been adopted to isolate tear components such as glucose, lactoferrin, lysozyme, and alcohol, for depicting the pathological states including dry eye symptoms, infections, diabetes, cardiovascular disease, endothelial dysfunction, coagulopathies, and polycystic ovary syndrome^6^. They have also been applied in testing breast cancer further suggesting the noninvasive diagnostic potential of tear exosomes^7^.

However, due to practical constraints in the isolation and handling of tear exosomes, despite their attractive potential, limited progress has been made in intensely acknowledging the clinical value of tear exosomes. Isolation is usually done on the principles relying on the size and particle density of exosomes. Tear exosomes isolation is commonly achieved by ultracentrifugation and size exclusion chromatography^8^. Ultracentrifugations’ efficiency is limited by its long processing time, large sample requirement, and low recovery while size exclusion chromatography lacks a proficient enrichment facility thus hampering the use of tear exosomes as diagnostic tools and discouraging further studies. Since the volume of tears samples collected from patients is usually scarce and the extraction procedure is complex, novel procedures are needed to be developed for paving new dimensions of disease deciphering by using tear as a sample.

Recently a team of researchers developed an incorporated tear-exosome analysis via a rapid-isolation system (iTEARS) platform to better comprehend exosome study and enhance exosome extraction^9^. iTEARS is a microstructure-based device containing a dual nanoporous membrane installed in a reservoir with two outlets operating on basis of a negative-pressure-oscillation strategy and on-device immunodetection. To ensure fast exosome isolation, alternate negative pressure switching is applied on both sides, and in this way, large particle exosomes get captured while small fragments (eg, proteins, sugars) are separated. This experiment exhibits that exosome can be enriched positively in under 5 minutes. The separated exosomes can then be utilized for different diagnostic analyses like PCR, sequencing, and proteomics. For practical utility of iTEARS, laboratory analysis of these exosomes was performed to distinguish a healthy control from Diabetes Mellitus and Diabetic Retinopathy. It was also used to identify different dry eye diseases thus emphasizing the important role of iTEARS and a hope for it discovering various other clinical conditions and their molecular diagnosis.

In the field of medicine, there is an ever occurring need to explore and identify new techniques for the diagnosis of disease and progression of pathology. Studies around human tear analysis have shown the potential to be a reliable and efficient method to present biomarkers for various pathological conditions of the human body. iTEARS technique not only produces results but is cost-effective and time friendly as well. Mega-scale clinical trials are needed to verify its potential for the diagnosis of disease. Tear sampling via iTEARS is feasible, noninvasive, easy to conduct, and effective.

## Ethical approval

Not applicable.

## Sources of funding

None.

## Author's contribution

All authors equally contributed.

## Conflicts of interest disclosure

The authors declare that they have no financial conflict of interest with regard to the content of this report.

## Research registration Unique Identifying number (UIN)

None.

## Guarantor

Syeda Shahnoor.
